# A theropod trackway providing evidence of a pathological foot from the exceptional locality of Las Hoyas (upper Barremian, Serranía de Cuenca, Spain)

**DOI:** 10.1371/journal.pone.0264406

**Published:** 2022-04-06

**Authors:** Carlos M. Herrera-Castillo, José J. Moratalla, Zain Belaústegui, Jesús Marugán-Lobón, Hugo Martín-Abad, Sergio M. Nebreda, Ana I. López-Archilla, Angela D. Buscalioni

**Affiliations:** 1 Departamento Biología, Paleontología and CIPb-UAM (Center for the Integration in Paleobiology), Facultad Ciencias, Universidad Autónoma de Madrid, Madrid, Spain; 2 Instituto Geológico y Minero de España, Madrid, Spain; 3 Departament Dinàmica de la Terra i de l’Oceà, Institut de Recerca de Biodiversitat, Facultad de Ciències de la Terra, Universitat de Barcelona, Barcelona, Spain; 4 Departamento Ecología, Ecología Microbiana, Facultad Ciencias, Universidad Autónoma de Madrid, Madrid, Spain; Birbal Sahni Institute of Palaeosciences, INDIA

## Abstract

We describe a trackway (LH-Mg-10-16) occurring in laminated carbonated limestones of the Las Hoyas locality, Serranía de Cuenca, Spain. It is unmistakably a large theropod dinosaur trackway encompassing two unusual aspects, namely, wide-steps, and a set of equally deformed left footprints (with a dislocated digit). The layer also preserves other vertebrate trails (fish *Undichna*) and different impressions in the sediment. To address these complex settings, we devised a multidisciplinary approach, including the ichnological and taphonomical descriptions, characterisation of the rock lithofacies using thin-sections, 3D structured-light digitalisation with a high precision of 200–400 μm, and a geometric morphometric comparison with a large sample of bipedal dinosaur trackways. Sedimentary analyses showed that the trackway was produced in a humid, benthonic microbial mat, the consistency and plasticity of which enabled the preservation of the details of the movement of the animal. The results of the geometric analysis indicate that the “wide-steps” of the trackway is not unusual compared to other trackways, providing evidence that it was made by a single individual with an estimated hip height approximately 2 m. Analogous pathologies in extant archosaurs that yield the combination of wide steps and deformed digits in the same trackway were considered. All results mutually support the hypothesis that a large theropod dinosaur, with a pathological foot, generated the trackway as it crossed an area of shallow water while slowly walking towards the main water source, thus stepping steadily over the benthonic mat over which multiple fish were swimming.

## Introduction

Pathologies in dinosaurs have been interpreted since the beginning of palaeoichnological studies. Hitchcock [1] mentioned a strange twist in the right footprint in four traces, and the same author deduced the absence of one of the toes in a didactyl track [2]. Later, Abel [3] cited *Eubronte* footprints with a broken digit II at the right pes. Since then, not many pathologies related to malformations have been reported from the Triassic to Cretaceous outcrops (Table 1). For example, Tucker and Burchette [4] described *Anchisauripus* footprints with malformations in toe II from the Upper Triassic (Norian) of Wales; Jenny and Josen [5] and Ishigaki [6] discovered theropod tracks with irregular digit morphologies from the middle Jurassic region of Morocco. Similar results were also cited in the Upper Jurassic of the Asturian coast [7]; and Currie et al. [8] quoted Cretaceous hadrosaur traces showing a bulbous expansion in toe IV. Other malformations inferred from the ichnological record were found [9–15]. All the cases listed refer to pathologies that produce a significant alteration in the footprint shape due to injuries, congenital, or biomechanical reasons (i.e., wounds, amputations). Furthermore, there are other pathologies, not necessarily in the footprints, that produce alterations to the trackway pattern. In limping dinosaurs, the trackway alternates between long and short steps, probably caused by illness, old age, and muscular or nerve injuries [16–18]. This alteration also involves laterality, that is, preference in the use of one limb over another, which, in some cases, generates a subtle difference in the length of the steps on both sides [19, 20].

**Table 1:** Pathological feet in dinosaurs. Theropod.

Data	Location	Age	Source	#im	Pathology
**1**	Portland Formation (Massachusetts)	EJ	[15], Fig 1	3	Loss of digit
**2**	Newark Supergroup	EJ	[15], Fig 2	2	Loss of digit
**3**	Lufeng Formation (China)	EJ	[13], Plate IC	2	Swelling digit II
**4**	Aganane Formation (Morocco)	mJ	[16], Fig 1	13	Limping
**5**	Morrison Formation (Utah)	LJ	[16], Fig 3	7	Limping
**6**	Lastres Formation (Spain)	LJ	[7], Fig 2	4	Trauma digit IV
**7**	Cabo Espichel (Portugal)	LJ	[17], Fig 1	11	Limping
**8**	Laiyang Group (China)	EK	[15], Fig 6	1	Loss digit II
**9**	Gates Formation (British Columbia)	EK	[18], Fig 7	11	Limping
**10**	Dakota Formation (Colorado)	mK	[15], Fig 8	1	Inward curvature digit II
**11**	Canada Goose (British Columbia)	mK	[15], Fig 9	4	Swelling digit IV
**12**	Kaskapan Formation (British Columbia)	LK	[15], Fig 10	1	Curvature digit III
**13**	Wapiti Formation (Alberta)	LK	[15], Fig 12	1	Dislocation digit IV phalanges
**14**	Wapiti Formation (Alberta)	LK	[18], Fig 8	3	Loss digit II

Summary of Jurassic and Cretaceous recorded tracks and trackways on pathological theropod footprints. Abbreviatures: #im, number of impressions, EJ, Early Jurassic, LJ, Late Jurassic; EK, Early Cretaceous, mK, mid Cretaceous, LK, Late Cretaceous.

Notably, there are few examples of complex pathologies that combine malformations and alterations in a long theropod trackway, because most of the reported ones are based on 1 to 3 imprints (Table 1). The trackway LH-Mg-10-16 from the upper Barremian locality of Las Hoyas [21] shows a deformation of one of the toes of the left foot and an unusually wide trace. The trackway, with six footprints, was previously published by Gibert et al. (see Fig 5 in [22]) and was identified as a theropod with strange movements. In this study, this trackway was reassessed expanding the evidences to understand the relationships between both alterations. The track was scanned and analysed metrically using geometric morphometric tools. The surface of the substrate incorporates more information about the traces themselves, such as scratches, folds, and other potential traces. We examined the footprints of the LH-Mg-10-16 trackway in light of the presence of a well-developed microbial mat, which is a characteristic condition of lacustrine sediments in the Las Hoyas wetland [21, 23]. We evaluated the extent to which the width of the trace could be due to a pathological pes or a taphonomic preservation produced in a shallow-water pond containing microbial mats. The final discussion is constructed based on crossing-hypotheses from ichnological approaches incorporating sedimentary, taphonomic, morphometric, and teratological evidence to reconstruct the rationality of the animal’s movement and its trackway.

### Las Hoyas locality

**Fig 1:**
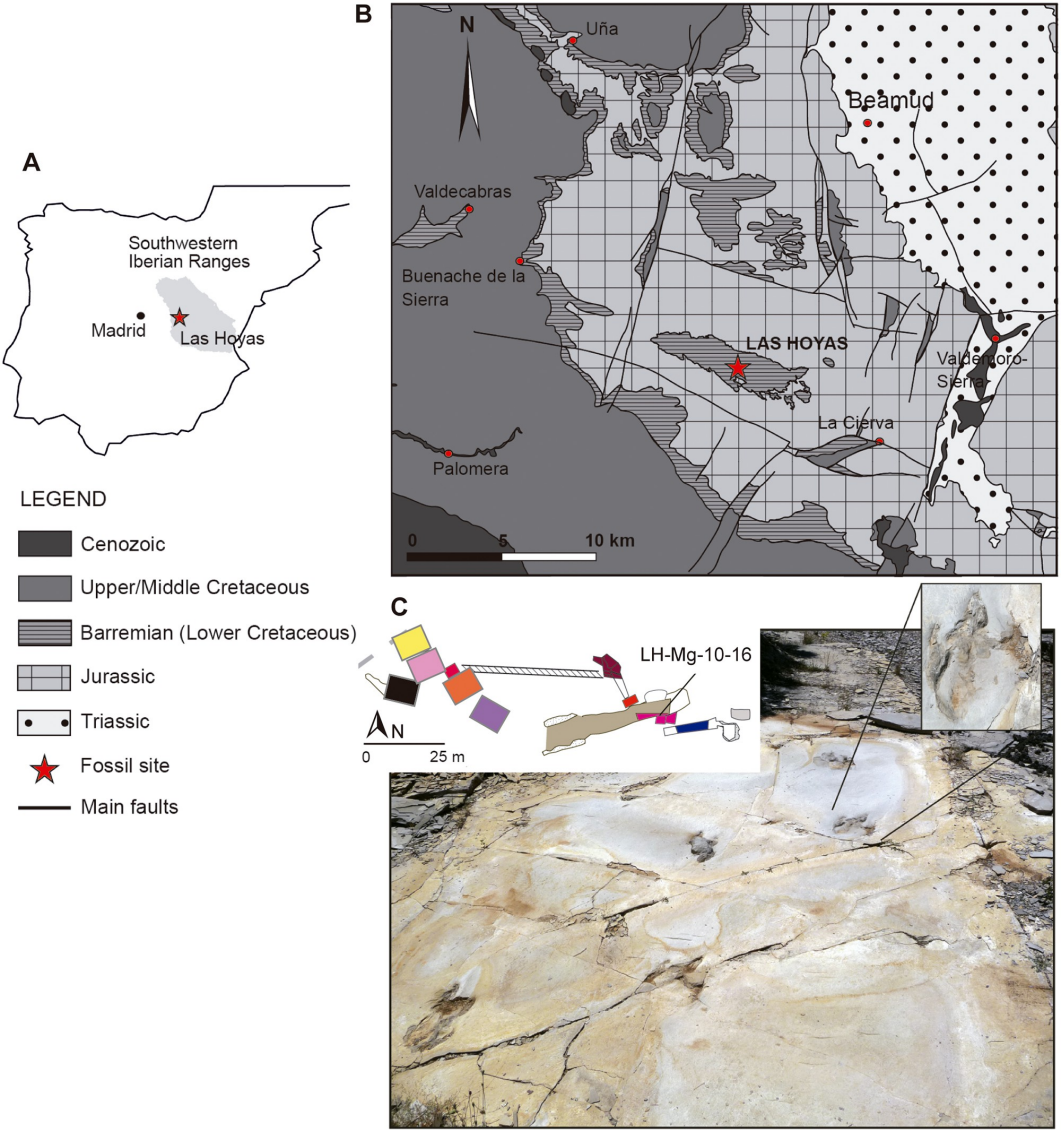
Geological settings and fossil site. A) Iberian Peninsula remarking the Southwestern Iberian Ranges where Las Hoyas locality (red star) is located. (B) Geological map of Serranía de Cuenca, depicting the Las Hoyas Basin. (C) Excavated squares and photography of the Theropod trackway and microbial impressions on the Magenta layer.

Las Hoyas is a small Basin within the La Huérguina Formation, which comprises the Barremian continental sedimentation in the southwestern sector of the Iberian Basin (Serranía de Cuenca, Spain, Fig 1A) [24, 25]. The Las Hoyas Basin (Fig 1B) was dated to 129–127 Ma on the basis of charophytes, ostracods, and palynomorphs [26, 27]. The Las Hoyas fossil site, with base coordinates: 40° 5’ 22”N, 1° 53’ 50.1”W, top coordinates: 40° 5’ 21.5”N, 1° 53’ 47.3”W, and elevation 1260 meters, comprises a 10 m lithosome of finely laminated limestones. It is well known because of the exceptionally preserved fossil bodies whose diversity ranges from algae to aquatic and terrestrial vertebrates [28]. Ichnology is represented by invertebrate and vertebrate traces [22, 29] as well as abundant coprolites [30]. Las Hoyas formed part of a regional inland wetland in the context of a carbonatic lentic ecosystem with a seasonal subtropical climate [21, 31].

The locality of Las Hoyas site was declared Paleontological and Historical Heritage since 2016 by the Government of Castilla- La Mancha. The site has been prospected since 1992, with the application of a systematic protocol of excavation, demarking squares of 25–30 m^2^ and proceeding layer by layer (Fig 1C). The set of excavated squares, named with colours, follows a stratigraphic succession from the east (Yellow Square) to the west (light Grey Square). The correlation of the squares was performed by combining the analysis of microfacies and the fossil content of each layer [21], as well as by connecting some sampled areas in the locality. The recorded tetrapod tracks become more prevalent towards the top of the stratigraphic succession, suggesting a change in the environmental conditions of the palaeoecosystem [29]. The magenta square was systematically excavated from 2005 to 2010. The magenta dinosaur trackway was discovered in 2010 within layer #16 (LH-Mg-10-16, Fig 1C).

## Material and methods

Field research has been carried out with the permissions of the Historical Heritage Department of the Government Junta de Castilla–La Mancha and Museo de Paleontología in Cuenca. SIFA action reference number: 16.1418.

### Ichnological material

**Fig 2:**
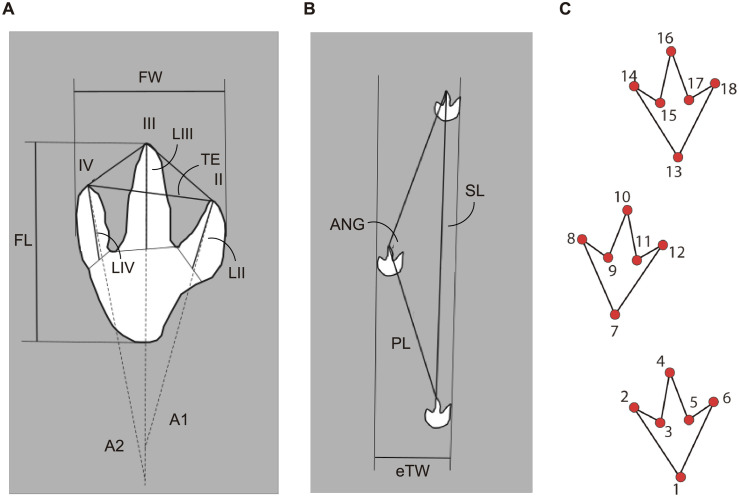
Metrics used. (A) Footprint measurements. (B) Trackway measurements. (C) Set of landmark used in geometric morphometrics. Abbreviations: ANG, pace angle; A1, angle between digits II and III; A2, angle between digits III and IV; eTW, external trackway width; FL, footprint length; FW, footprint width; LII-IV, length of digits; PL, pace length; SL, stride length; TE, toe extension.

The footprint contours of the dinosaur trackway (LH-Mg-10-16) are already sketched in Layer #14. Layers #14 to #16 are characterised by the presence of abundant fish trails and a relatively small number of fossils (less than ten, comprising plants and insects). The magenta LH-Mg-10-16 recorded six isolated footprints and pertained to a rather large trackway. To describe the taphonomy and footprint shape, we followed the terminology used by Marty et al. [32], which differentiates the features that came from the interaction of the animal and sediment (i.e., true track, track wall, displacement rim, overall track, track surface, and underprint) from the features related to the process of burial (sediment fill, over track, undertrack). The linear measurements of the trackway and footprints follow the methodologies proposed by Moratalla et al. [29] and Costa-Pérez et al. [33] for geometric morphometry (Fig 2).

### Thin sections

To study the microbial mats, two blocks of rocks were separated from the magenta square, the base of the upper block (T1), and the top of the block beneath (T2) corresponded to the trackway layer. Each block was 5–7-cm-thick. Two thin sections were cut from these blocks with the following dimensions: 43 mm width × 55 mm length. Then, they were photographed with an OLYMPUS Tough TG-5 camera and a Motic BA310 stereomicroscope.

### Structured-light 3D scan

**Fig 3:**
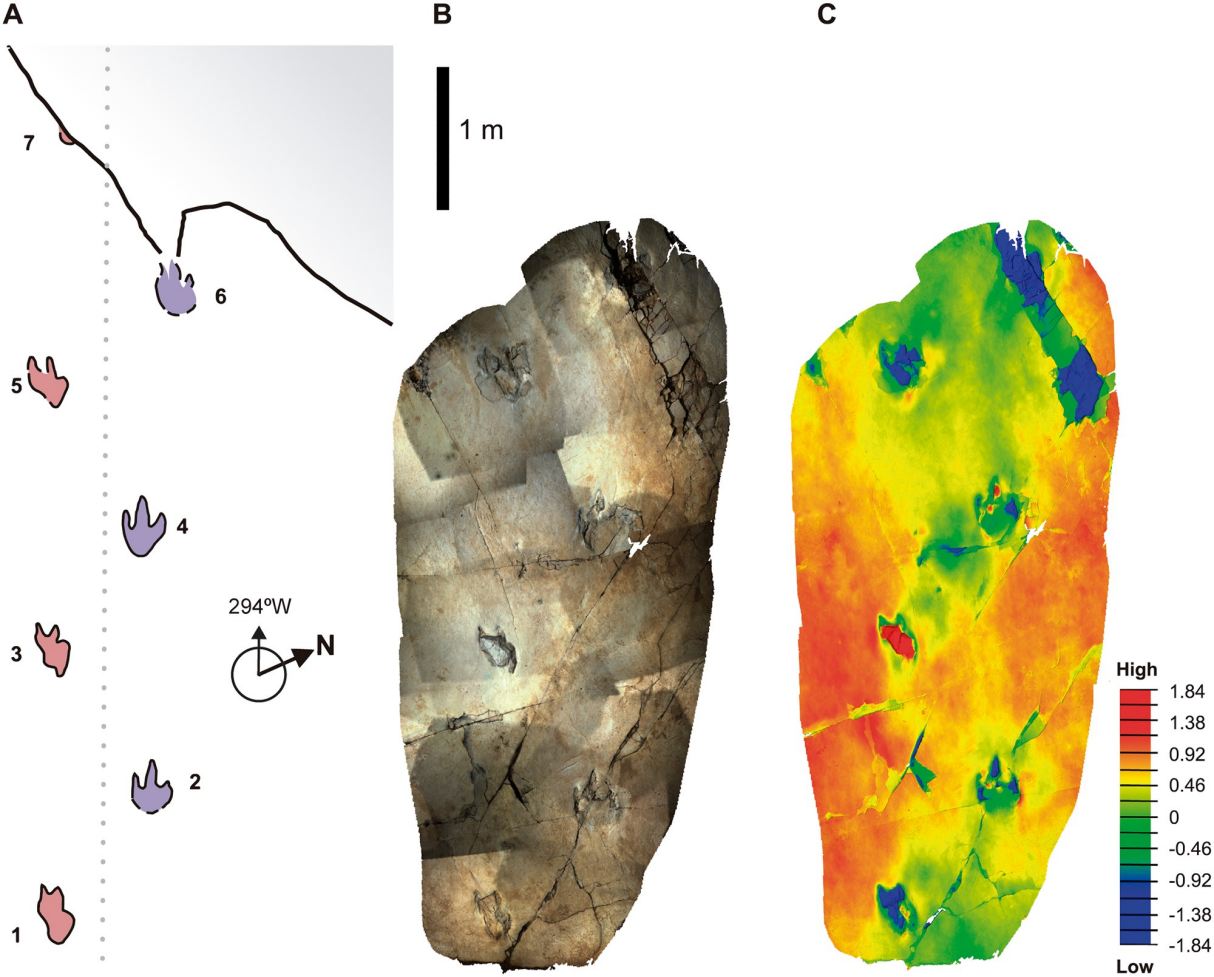
Magenta LH-Mg-10-16 trackway. (A) Cartography done in the field, (B) Scanned surface of the trackway, mounted from a set of color photos. (C) Color ramp, showing the depths in the legend (in cm). Scale, 1 meter. The cartography is reprinted from Las Hoyas a Cretaceous wetland, Gibert JM et al. [22] under a CC BY license, with permission from Dr. Friedrich Pfeil Verlag, original copyright 2016.

Because the trackway will remain at the locality, a 3D model is essential to preserve the fossil and its heritage value [34]. The dinosaur trackway surface occupies 5 m × 1.4 m (Fig 3A), and it was scanned in 2018 by the *Paleoymas Corporation* (Fig 3B and 3C). The area was mapped using a Breuckmann SmartScan 3D HE-color scanner, which is a non-invasive procedure consisting of a structured-light 3D scanner with a precision of 200–400 μm with 3D high-resolution white light. The scanner was calibrated using geometrical patterns for more than an hour. The whole scan took place from 02:00 to 08:00 hours to avoid the interference of daylight. Using this high-resolution scanner, complete information of the trackway and sedimentary layer could be stored digitally with a precision of micrometers (https://www.aniwaa.com/product/3d-scanners/aicon-3d-systems-breuckmann-smartscan/). Because of the extent of the trackway, scanning was performed in multiple steps, adding the different scans into one unique file, and adjusting their relative size and position with respect to what was measured in the field with an overlapping index of 30–40. After processing the data obtained and conducting strict quality control tests on the level of overlapping, a dense cloud of vertices with a minimum resolution of 0.0004 m between vertices was obtained. The data were stored in the form of a polygon file format.ply, defining a virtual 3D mesh composed of vertices and faces with a locked relative position (3D model available in the open access repository Morphosource, https://www.morphosource.org/concern/media/000410057). Additionally, with each scan, photos were taken to ensure that each point of the vertex cloud was associated with the real colour of the track (S1 Fig).

**Fig 4:**
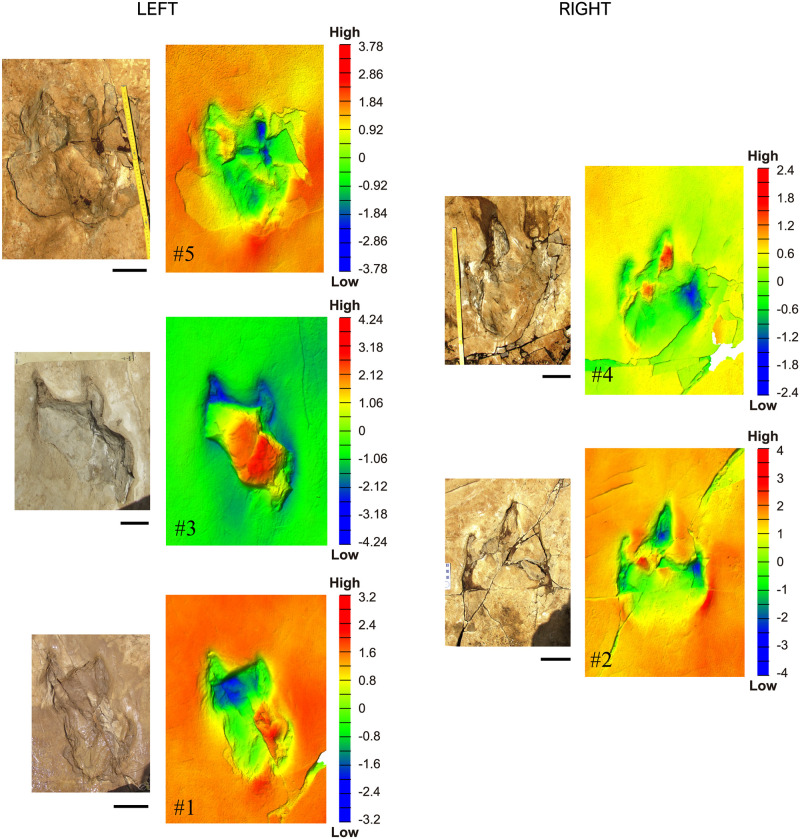
Magenta LH-Mg-10-16 left and right footprints. Footprints ordered from # 1 to # 5, pairing the photograph with the scan and its color ramp (legend in cm). Scale, 10 cm.

To study the layer topography of the surface, the inclination was corrected to a horizontal plane (x–y), dropping the layer dip angle from 10° to 0°. The edited mesh was adjusted to a colour ramp along the z-axis to observe the relief of traces in detail using MeshLab [35] and CloudCompare [36]. The common gradient used for surface analysis involves red-yellow-green-blue colours, with red being the highest point in the z-axis, and blue being the lowest. The maximum and minimum points considered for the colour gradient were established depending on the feature of interest, such that, every footprint and sedimentary structures (cracks and wrinkles) were independently analysed and represented with their own colour ramp (Fig 4).

### Geometric morphometrics

The magenta trackway was compared to a sample of 75 bipedal dinosaur trackways taken from the literature [33]. Accordingly, six landmarks (Fig 2C, Table 2) were selected to capture the most salient features of the three footprints within the trackway following the protocol devised by Costa-Pérez et al. [33]. Three footprints (i.e., one stride) are the minimum required to describe a trackway [37]. Thus, the entire magenta trackway was split into two subsets of two strides (Fig 3).

**Table 2:** Landmark description.

Landmarks	Anatomical equivalence
**1, 7, 13**	Heel
**2, 8, 14**	Tip Digit II
**3, 9, 15**	Hypex II–III
**4, 10, 16**	Tip Digit III
**5, 11, 17**	Hypex III–IV
**6, 12, 18**	Tip Digit IV

These landmark coordinates are superimposed following a generalised Procrustes superimposition which is based on the least squares criterion [38, 39]. The residual variation left by the superimposition is devoid of (and invariant to) translation, rotation, and scale, and is referred to as ‘shape’. Because it is multidimensional, it is best summarised using principal component analysis (PCA). The Procrustes superimposition is sensitive to bilateral symmetry [40], facilitating the comparison of the tracks irrespective of whether they start from one foot or another.

## Results

### Descriptive ichnology

#### Trackway LH-Mg-10-16

Trackway LH-Mg-10-16 consists of at least six tridactyl dinosaur footprints (Fig 3A). The direction of the trackway was approximately 294° W. The trackway shows a wide-step pattern and deformed footprints. The footprints are preserved as concave to convex epireliefs, and only one footprint (#3) is a clearly convex epirelief, preserved as an over track of a stack of internally laminated sediment. The footprints have relatively acuminate digital prints, a V-shaped digit III, a narrow interdigital angle, a narrow and elongate “heel” surface, and an asymmetrical shape.

The trackway shows a significant difference in morphology between the left and right traces (Fig 3). The footprints on the left side are slightly shorter (mean footprint length of 35.33 cm versus 44 cm for the right side, Table 3) and more irregular in shape (digit II is almost missing, very short, distally rounded). Digits III and IV on the left side are approximately of the same relative length, and the heel surface is irregular (Figs 3 and 4). The medial margin is continuous, while the lateral outline shows an irregular pattern with a single (footprints #1 and #5) or even a double indentation (footprint #3) (Figs 3 and 4). The right footprints are more regular in shape (Figs 3 and 4). They show relatively acuminate digit prints, a V-shaped digit III, relatively narrow interdigital angles, a narrow and elongated heel surface, and an asymmetrical general morphology. The right footprint #6, is very deformed and is partially broken in its anterior zone.

**Table 3:** Footprint variables.

#Tr	FL	FW	iFL	rFL	iFW	rFW	LII	LIII	LIV	TE	A1	A2	R1	R2
**1**	36	25	36	-	25	-	-	9	6	9	-	-	1.4	0.4
**2**	43	32	-	43	-	32	6	20	7	19	22	21	1.3	0.3
**3**	34	26	34	-	26	-	3	12	11	10	-	-	1.3	0.3
**4**	45	29	-	45	-	29	6	18	5	13	20	19	1.5	0.5
**5**	36	29	36	-	29	-	1	14	8	10	-	-	1.2	0.2
**6**	-	-	-	-	-	-	-	-	-	-	-	-	-	-
M	39	28	35	44	27	30	4	15	7	12	21	20	1.4	0.4

Measurements of each footprint of the Magenta LH-Mg-10-16 trackway taken in field. Abbreviations: #Tr, track number; FL, footprint length; FW, footprint width; i, referred to the left footprints; r, referred to the right footprints; LII, length of digit II; LIII, length of digit III; LIV, length of digit IV; M, mean; TE; toe extension; A1, angle between digits II and III measured from the rear margin of the heel; A2, angle between digits III and IV measured from the rear margin of the heel; R1, ratio length-width of the footprint FL/FW; R2, footprint ratio FL-FW/FW. Labels used as in Fig 2A.

The non-deformed footprints depict relatively elongated and narrow feet (FL/FW = 1.44), showing subparallel and almost aligned digits with low interdigital angles (II–III, 21° and III–IV, 20° as mean values), relatively similar lengths of digits II and IV (although toe II appears to be slightly longer and slenderer), and a high degree of mesaxony (TE/FL = 0.31) with a noticeably V-shaped morphology of digit III. The altered footprints result in similar dimensions of digits III and IV, and in an altered footprint length between the tip of digit III and the posterior margin of the heel.

The trackway pattern is peculiar in its width (Table 4), as there is a clear internal separation (approximately 45 cm) between the left and right footprint sets. The hip height (H), using the method of Thulborn [37], was estimated to be 190 cm. Stride and hip height were subequal (SL/H = 1.03). Its stride length is approximately five times the foot length, according to the mean footprint length (SL/FL = 5), whereas the external trackway width is approximately four times the foot width (eTW/FW = 4.11). Metrics based on the hip height (approximately 190 cm) suggest an animal body length of 6–7 m. The trackmaker was walking at a relatively slow speed of v = 4.06 km/h.

**Table 4:** Trackway variables.

#Tr	SL	PL	ANG
**1–3**	193	-	106
**2–4**	200	-	109
**3–5**	-	-	111
**1–2**	-	115	-
**2–3**	-	123	-
**3–4**	-	122	-
**4–5**	-	129	-
**5–6**	-	-	-
M	196.5	122.2	108.6

Variables of the Magenta LH-Mg-10-16 trackway. Abbreviations: #Tr, track number; SL, stride length; PL, pace length; ANG, pace angle; M, mean values.

#### *Undichna* traces

**Fig 5:**
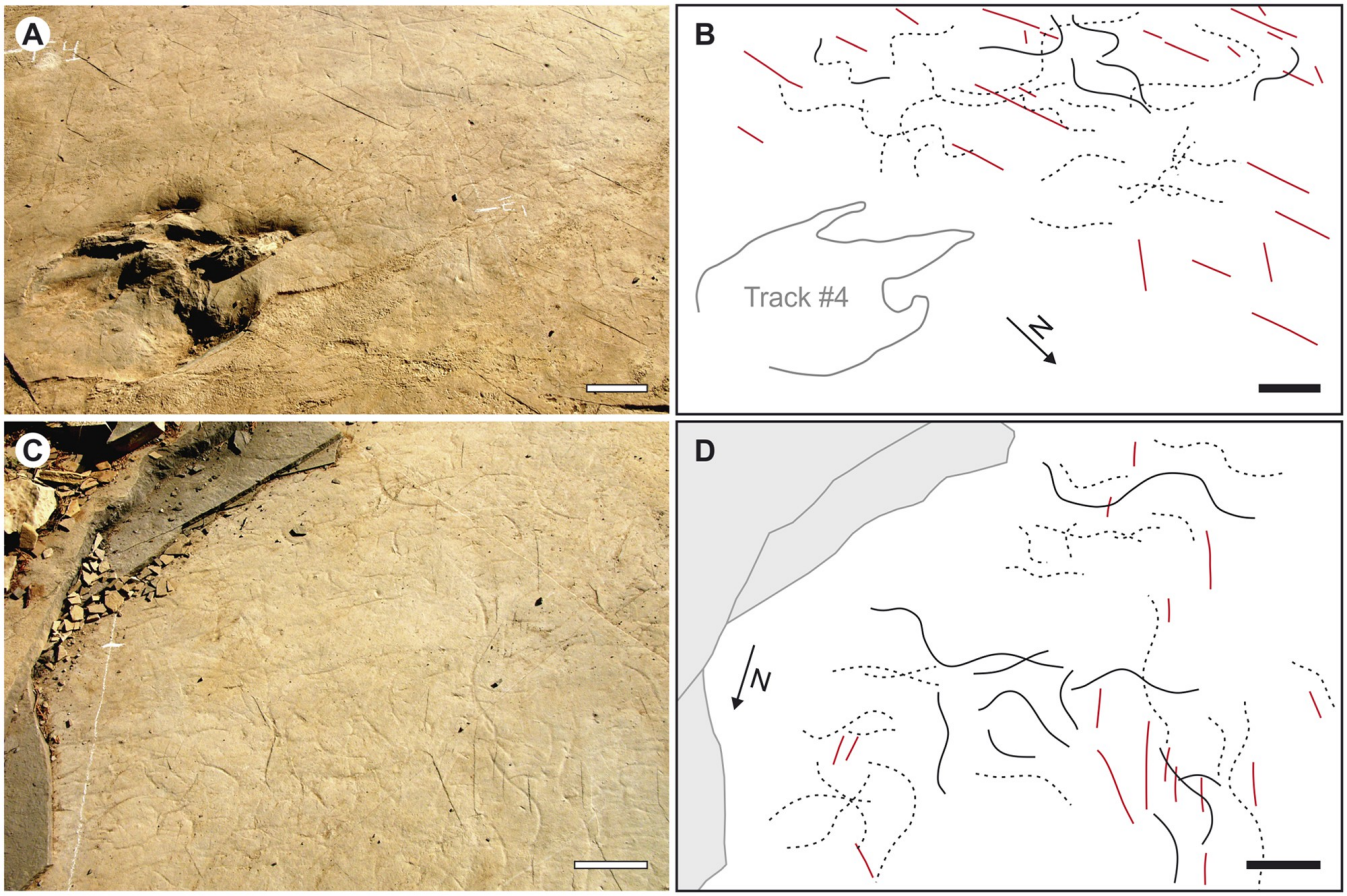
*Undichna* fish trails in Magenta LH-Mg-10-16. (A) and (B). Fish trails associated to the theropod footprints. (C) and (D). Accumulation of fish trails. Lines in black trails, and in red, wrinkles marks. Scale, 10 cm.

Sharply incised grooves, preserved as concave epireliefs, and having the form of a single sinusoidal or slightly asymmetrical sinusoidal wave are present on the same surface on which the studied trackway is located (Fig 5). These trace fossils are the most abundant on that surface, and hence, a frequent and complex crosscutting relationship among specimens is observable. Nevertheless, it is possible to identify 10 to 20 cm traces of complete waves.

The morphological features of these trails are diagnostic of the ichnogenus and ichnospecies *Undichna unisulca*, an ichnotaxon erected by [41] at the fossil site of Las Hoyas. Interpreted as fish swimming traces and taking into account the body-fossil record of Las Hoyas, Gibert et al. [22, 41] proposed pycnodontiforms as the most likely trail makers. Nonetheless, the relative abundance of *Undichna* traces contrasts with the low abundance of pycnodontiform fossil bodies in comparison with other common fish groups in the locality.

### Substrate and the microbial mats

**Fig 6:**
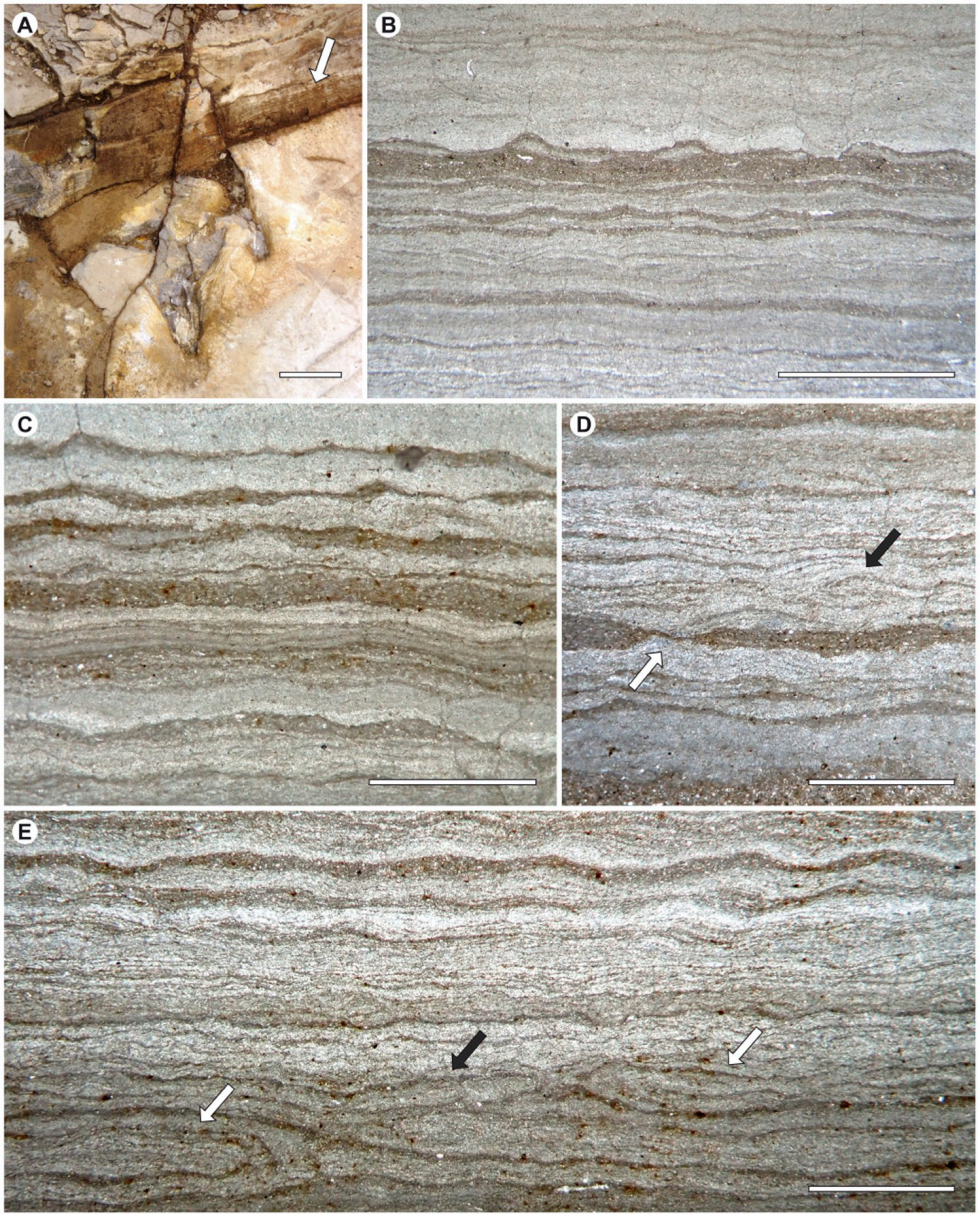
Magenta layer biolamination. (A) Lamination of limestones and footprint. (B) and (C) Wavy-crinkly structures of the internal lamination. (D) and (E) Overfolded mats, showing ‘tear in mat’ process (black arrows), and convolutions (white arrows). Scale bar in A, 10 cm; scale bar in B-E, 1 mm.

The fine lamination that characterises the limestone studied herein (Fig 6) has been identified as biolamination or MISS (i.e., microbially induced sedimentary structure, see [42, 43]). This most likely originated from a flat microbial mat. A microbial mat is formed by a community of microorganisms, primarily comprising phototrophic filamentous cyanobacteria, anaerobic photobacteria, and other diverse types of prokaryotic and eukaryotic microorganisms [44]. The community is disposed of in horizontal layers that grow vertically in relation to light and oxygen requirements [45].

Schieber [46] enumerated a set of sedimentary features indicative of the presence of microbial mats. From the thin sections of Las Hoyas limestone, it has been possible to identify: (a) the wavy-crinkly character of laminae (Fig 6B and 6C); (b) the presence of carbonaceous laminae (Fig 6B–6E); (c) overfolded mat layers showing the cohesive behaviour of laminae (Fig 6D and 6E); (d) fragmented and slightly convoluted mat layers (Fig 6D and 6E); and (e) structures comparable to the result of a ‘tear in the mat’ process (Fig 6D). Additionally, the texture and rugosity of some areas of the studied surface also indicates the presence of microbial mats.

According to the environmental conditions, microbial mats may develop at the sediment-water interface and/or float on the surface of standing bodies of water [47]. In the case of the Barremian limestone of Las Hoyas, both the exceptional preservation of fossil bodies and the identified sedimentary features point to the benthonic development of these microbial mats (see also [45, 48–51]).

**Fig 7:**
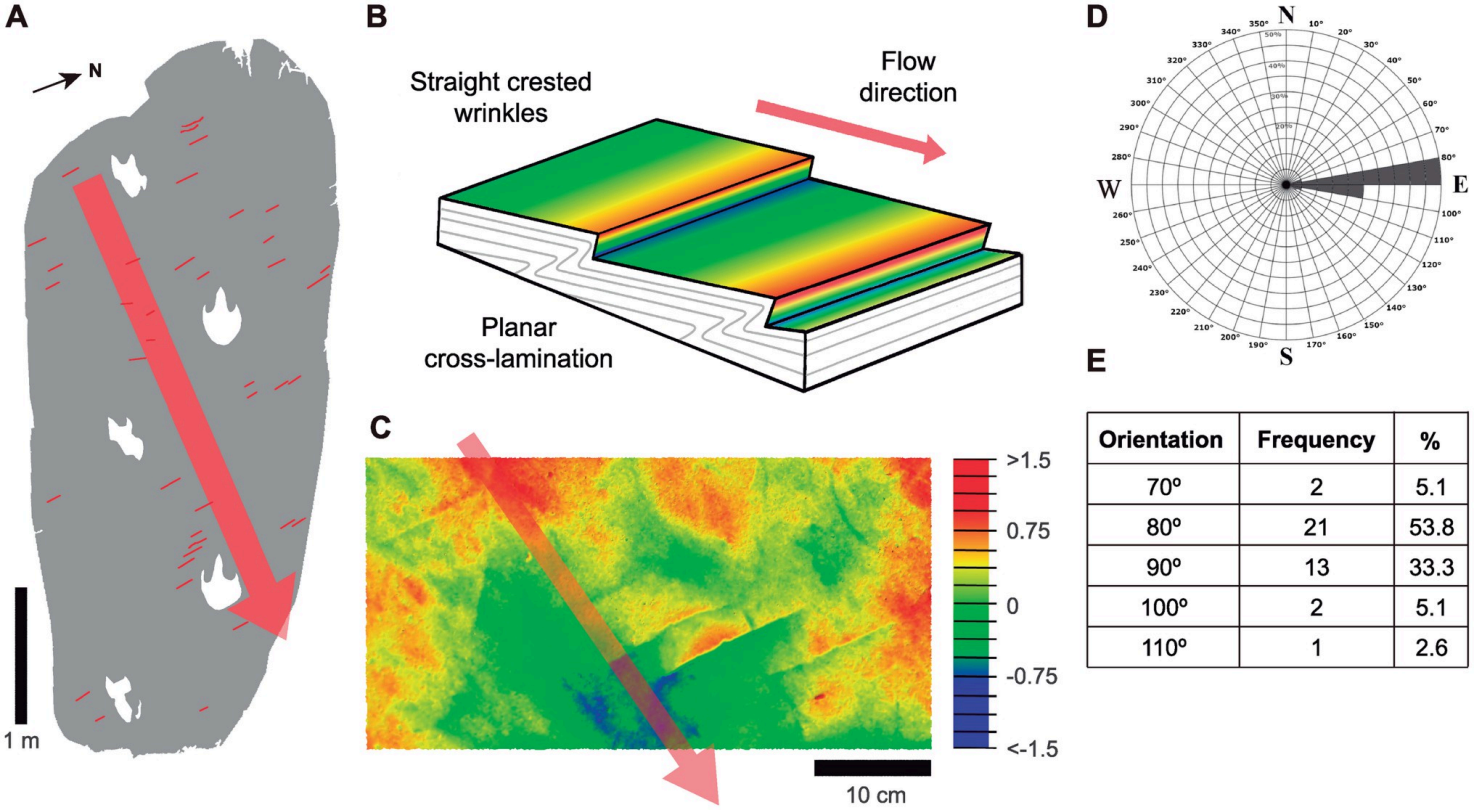
Magenta LH-Mg-10-16 surface structures. (A) Distribution of wrinkles (in red) on the layer. (B) Schematic view of the wrinkles following the analogy of ripple marks formation. (C) Wrinkles with color ramp and depth scales in cm. The red arrow indicates the direction of the flow. (D) Rose diagram showing the resultant current flow orthogonal to the fold orientations. (A) Layer #16 with the distribution of wrinkles in red. (B) Schematic view of the wrinkles following an analogy of ripple marks formation. (C) Wrinkles with color ramp and depths (scale in cm). (D) Rose diagram showing the resultant current flow, orthogonal to the wrinkle orientations. The red arrow indicates the direction of the flow.

Notably, we recognised groups of wrinkles over the same layer where the dinosaur walked (Fig 7A). The wrinkles are similar to ripple marks, being parallel, short (ca. 25 mm), unequal, and present in sets of three or four (Fig 7B). These thin wrinkles, probably related to the growth of microbial mats [52, 53], form patches that are sparse and intermittent. The creases have a subtle and progressive increase in height and an abrupt drop after the crest; the progressive height increase is always positioned on the same side as the crest. The creases show a preferred orientation (Fig 7C–7E), quasi-parallel to the north, indicating the flow direction as the resultant perpendicular.

### Multifaced approach to the problem: Taphonomy and morphometrics

The ichnological record of tetrapod footprints at Las Hoyas exhibits a disparity in preservation ranges (from blurred traces to those with sharp contours). In fact, footprints in lacustrine wetlands mediated by microbial mats often have mediocre preservation [29, 32, 54]. Our overall goal was to understand the disparity of vertebrate ichnofossils in relation to the environmental and abiotic factors of the ecosystem. Previous outcomes [29] led to the identification of two groups of tetrapod traces in Las Hoyas: (1) shallow prints that reflect the gross outline of the feet, and (2) deep, traceable prints that show a more detailed impression of the toes. In particular, the magenta theropod trackway LH-Mg-10-16 falls within the second group, deep prints preserving the foot contour, wherein the foot contours are especially unequally conserved throughout the trackway.

The peculiarities of the magenta dinosaur trackway (Figs 1, 3 and 4; Tables 3 and 4) prompted us to verify a set of questions in order to understand how and why an abnormal external trackway width and asymmetrical footprints can be produced in the same trackway. We focused on determining the asymmetry of the foot shapes: whether the deformed footprints were due to taphonomic factors (e.g., footprints produced on a slick surface), or whether they were due to pathology and/or individual malformation. Because of the wide trackway gauge, we tested whether it was a single trackway, or if LH-Mg-10-16 corresponded to two parallel trackways. Finally, we reconstructed the animal movement in the context of the Las Hoyas lacustrine calcium carbonate wetland ecosystem.

#### Taphonomy of the studied tracks

The footprint outlines are clearly recognisable as dinosaur traces, and all maintain similar proportions (Table 3), although the left and right footprints show different lengths (Fig 4). The overall track of each print includes the true track, displacement rims, especially lateral and posterior, and occasional claw imprints. The base of the true tracks cannot be directly observed because footprints are partially filled with sediment, but the heels are level with the tracked surface.

**Fig 8:**
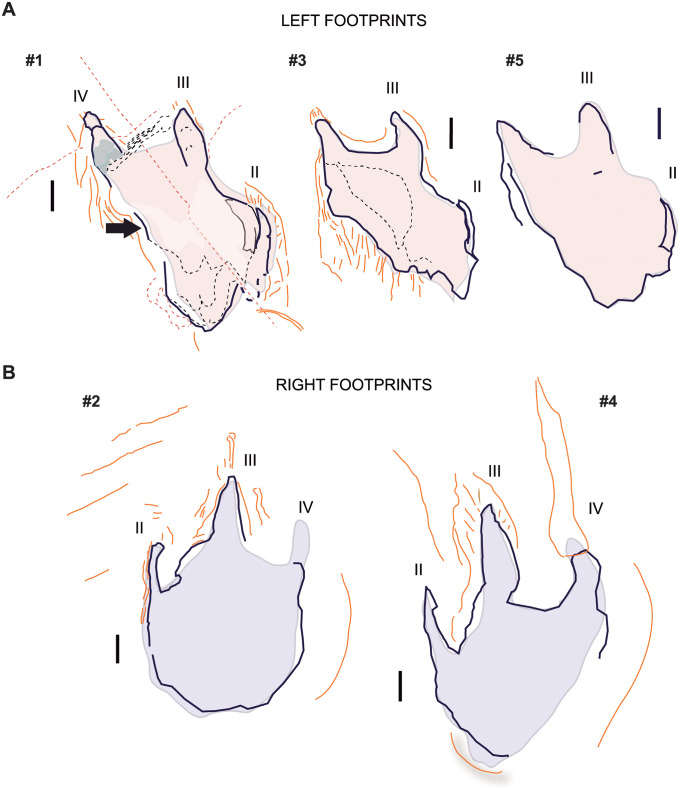
Magenta LH-Mg-10-16 taphonomic features. (A) Left footprints. (B) Right footprints. Black lines corresponds to unambiguous limits, whereas the filling color (red or violet) reflect the overall impression. Lines in orange describe marks around the track (i.e., bulged areas, nail scratches, stepped borders of the contour). Dotted lines correspond to breakages, or discontinuities. The arrow points to the medial indentation. Drawn from photographs of isolated footprints. Scale, 5 cm.

The prints have high track walls corresponding to the deformation of the tracked surface layer. The deepest walls were observed in the digital arch. Digits are filled with sediment, which is roughly laminated, forming internal over tracks. Digit imprints preserve a thin crust that delimits contours. In one of the traces (footprint #1), the foot crossed a pile of laminations observed in a broken section of the layers, exposing a hardened substrate between the digits (Fig 8A). No under tracks were observed. Striation traces are observable and were produced by: (a) claws; (b) radial fissures at the rear of the footprints associated with a displacement, (c) rims at the rear of the footprints, and (d) bulked areas around the footprint due to the deformation of the substrate by the animal weight. The left footprints (# 1, # 3, and # 5) present an intense layer deformation compared to the right footprints (# 2 and # 4).

The left footprints are preserved as almost complete outlines; they expose stepped contours (Figs 4 and 8) mostly visible at the lateral contour that shows a succession of sediment deformations, particularly at digit II (Fig 8A, see footprint # 1). Indentation appears posterior to the denominated digit IV. The medial outline shows a depressed semilunar groove, which is filled with sediment, similar to the grooves of other digits. The groove was interpreted as digit (II). At the contact between this groove and the palmar area, the sediment copied a lobe. The posterior footprint region is elongated, and the sediment copied a mid-furrow (Fig 8A, see footprints # 1 and # 5).

The right footprints are more regular in shape. The right footprints are characterised by a stepped central and medial digit printing, particularly by a lateral bulked area. A fingernail print was produced in the resulting step in footprint # 4 (Fig 8B). The traces have different contours due to sediment collapse or infilling, and the ‘heel’ is at the level of the printed layer (Figs 4 and 8).

#### Deformed foot

**Fig 9:**
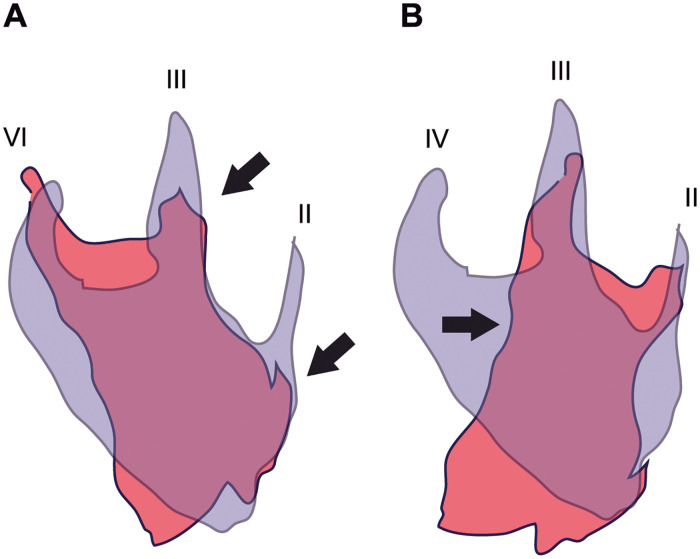
Hypotheses on left footprint deformation. Superimposition of left (red) and right (violet) footprints. The violet contour has been horizontally rotated, for a same digit comparison. The arrows mark the main features that should be considered to interpret the deformation (see text). (A) Digit II damaged is the preferred hypothesis. (B) Loss of digit IV.

The front projection of digit II is completely missing, and the mark of this digit is likely backwardly displaced (enhanced in left footprints #1 and #3, Figs 4 and 8). The injury and/or loss of digit II, which has been interpreted as the most plausible hypothesis (Fig 9A), presumes that: (a) digit III should be thicker but also reduced or affected in length; (b) the interdigital areas are wide in both digits II and IV; (c) digit II is slender and has lost some phalanges; (d) it is twisted and only a splint would be preserved and impressed; (e) digit IV should be stout; (f) the lateral indentation would be an artifact; and (g) the pes would not be rotated and the three digits forwardly directed. A rotation of the foot while walking would leave the signs of oblique striations, however, the scratches (footprint # 3) are in the same direction as the trackway.

In comparison, the alternative hypothesis entailing the injury of digit IV ought to consider the lateral indentation (i.e., the arrow in Fig 9B), and assume that it represents the proximal end of the broken digit IV. In this interpretation it must be supposed that the presence of a digit I impression as a rod, placed medially and posteriorly and connected with the ‘heel’ foot area (Fig 9B). Then, (a) digit III would be the largest and longest; (b) the interdigital areas between digits would be wide; and (c) digit II would be rather short. With the loss of digit IV, the shape of the footprints would be stretched and rotated, and the plantar surface would be medially broadened due to the incorporation of digit I. Importantly, the hypothesis would require the lateral rotation of the foot in the trackway, thus distorting and increasing the width of the angulation pattern (measured perpendicular to the stride length), as well as the rotation of the trace with respect to the next stride line (see S1 File). Because so many assumptions were needed for the hypothesis of the loss of digit IV, it was rejected. Other unlikely explanations for the deformed foot are addressed in S1 File.

#### Trackway pattern and geometry

**Fig 10:**
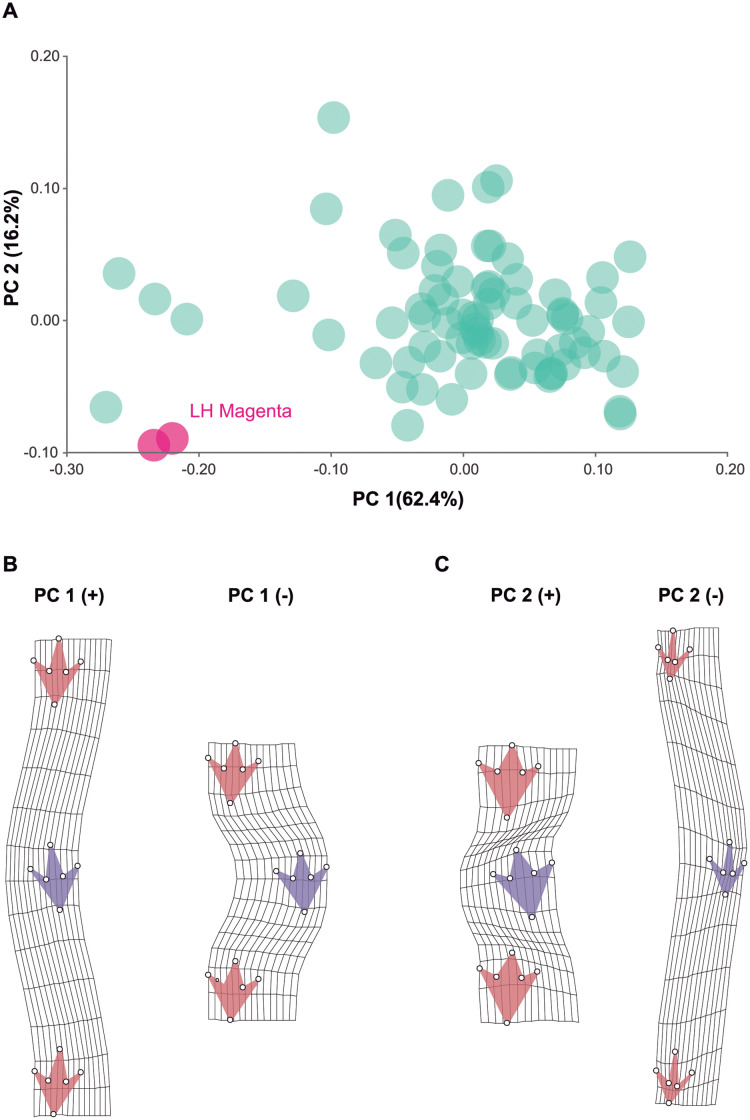
Magenta LH-Mg-10-16 PCA of geometric morphometric analyses. (A) Comparison of the two trackway subsets of Magenta with other bipedal dinosaurs worldwide. (B) Shape transformations in PC1 and (C) Shape transformation in PC2.

The landmark configurations were digitised according to the accepted hypothesis on the deformed foot, wherein the trackway shows a strongly parallel and frontal disposition of the right and left toes (Fig 3B). It would be very difficult to digitise landmarks on each side alone; on the right, there are only two well-preserved tracks, whereas digitising the left side alone would yield uniquely deformed footprints. Thus, two subsets were needed to complete the entire trackway (Fig 10A). However, the two subsets only have subtle differences compared to a randomly selected sample of bipedal dinosaur trackways. Shape Analysis (GM) unambiguously shows that the two subsets of footprints (Fig 10A) belong to the same trackway, supporting the hypothesis that the footprints belong to a unique trackway. Furthermore, the position of the Magenta trackway in the scatter indicates that it is more similar to a small group of bipedal trackways that differ from the great majority, which are clustered much closer to the mean (i.e., those that are statistically normal). Their scores, greater than -0.20 at PC 1, correspond to very large Procrustes distances from the Mean (0.0), meaning that they are very different. As such, the magenta trackway spans a relatively ample external trackway width and has smaller steps, but this is a common feature among such outliers. Interestingly, such grouping encompassing outlier “wide-stepped” trackways were deemed ornithopod-like in the literature, but shape analyses failed to corroborate this assumption [33]. In addition to the latter major differences captured by PC 1, what makes magenta trackway slightly different from previous records of trackways is that it shows relatively longer steps with relatively smaller and slender footprints (PC 2, Fig 10C).

## Discussion

The main link to be proven in the study of the magenta LH-Mg-10-16 theropod trackway is that it was produced by a single animal. The ichnology and morphometric approaches ratified this statement. The morphology of the footprints on the right and left run along parallel lines, and their distribution is clearly specular, compatible with the hypothesis that the footprints of the left side were produced by the left foot of the animal, and the right foot made the footprints on the right side. Shape analysis substantiates the equivalence of two strides belonging to the same trackway and demonstrates that the amplitude of this trackway is characteristically wide, comparable to that of a handful of trackways that were identified as an ornithopod, instead of theropod, for the same reason [33].

Frequently, theropod dinosaur trackways are narrow, with longer strides and relatively high pace angles [33]. The overall pattern of LH-Mg-10-16 is peculiar, including a high width and low pace angle, for a bipedal dinosaur trackway (Fig 10B and 10C). Likewise, the orientation of the tracks with respect to the trackway’s midline is slightly different from the frequent inward rotation of bipedal dinosaur trackways. However, despite this morphology, the magenta trackway maintains (1) a regular and relatively constant stride length (mean value: 196.5 cm), suggesting a consistent and relatively constant speed, and (2) a footprint rotation of nearly 0°. The trackway shows a direction of movement of approximately 294° W, being quite straight, while maintaining a consistent stride. This suggests that the walking speed is relatively constant, with a regular walking pattern.

### Pathological evidences

The peculiar features—a trackway with wide external steps and the footprint’s left and right asymmetry—of the magenta #16 layer are explained as a result of foot pathology. Despite this deformity, the geometry of the trackway suggests that the animal laid its foot completely on the substrate, so that even the depth of the left footprints seems to be fairly constant, with no significant variations in the different areas. Surprisingly, the trackways showing a limping pattern, as observed by Lockley et al. [16], do not seem to show pathologies in their tracks. For instance, the same occurs in the trackway of a theropod dinosaur from the Lower Cretaceous period in Canada [15], where, although the left footprints are strongly rotated inwards, the trackway pattern appears relatively normal.

In addition, because extant archosaurs show similar pathologies, combining the widened tracks and toe deformities, we suspect that the left foot pathology also influenced the amplitude of the trackway. Birds suffer with so called ‘crooked toes’ or ‘curled toes’ [55–57], as has been studied in poultry and domestic ostriches [58]; a deformity related to genetics, environmental factors, or dietary deficits [55, 58–61]. Abnormal gait due to injury (leg fracture) has been described in the waterbird Scolopacidae [62] by detecting a reduction in toe footprints and asymmetric steps. Although few cases have been studied in wild birds and crocodiles, osteopathologies with frequent foot bone fractures have been documented in theropods; in these, digit II is often the one with the greatest number of incidences, including swelling, torsion, and backward disposition [15, 63].

### Moving animal scenario

To justify the plasticity of the substrate, its rapid lithification, the filling of the footprints, and the presence of a crust around the toes, it was necessary to verify the presence of a microbial mat that makes up the lithofacies of the Las Hoyas limestones [23]. Most of the known microbial mat communities are associated with sediment-water interfaces. A living microbial mat normally prevents sediment desiccation, which in turn promotes an active lithification process with carbonate precipitation [64]. Phototrophic filamentous cyanobacteria play a key role in capturing sedimentary or suspended particles of various sizes, and under certain conditions, precipitation and lithification are favoured within the community by sulfate-reducing bacteria [65, 66], anoxygenic phototrophic bacteria [67], and diatoms [68], among others.

**Fig 11:**
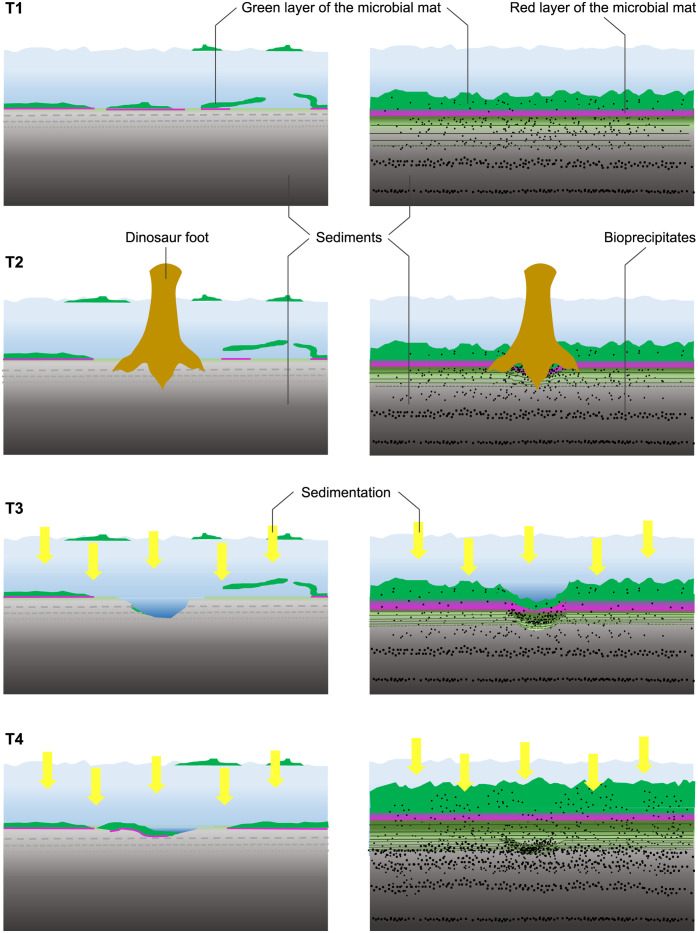
Formation of footprints in microbial mats in a shallow column of water. The left column represents a simple, slim and slightly floating mat. The right column considers a complex and consistent benthic mat. The animal foot tearing the mat, would have printed the bottom substrate. On the left sequence there would not be vertical continuity between the mat and the substrate, and traces would have had imprecise limits. On the right, the foot would deform the surface, and the mat layers would slide towards the inner footprint surface. The compression of the mat would favor the concentration of the inorganic particles crucial to biomineralize and lithify the substrate.

The mat communities are not so variable as to account for the abnormal width of the magenta pathway, as the mats never expand laterally, but instead build horizontal layers that grow vertically in relation to physical and chemical conditions. The Las Hoyas #16 layer suggests a wet plastic mat that favoured the production of deep tracks. In addition, a thick and consistent mat would be required to support a mobile 5 ton dinosaur without breaking the continuity of the mat surface (Fig 11). The lithification process would have occurred steadily since the underlying layers of the traced surface were hardly deformed. Lithification, on the one hand, would favour the preservation of many details in LH-Mg-10-16 related to animal movement, such as the marks and deformations observed in the right footprint that suggest a greater load on its right foot, indicating a slight antalgic gait. On the other hand, the continuous growth of the mat is capable of blurring parts of the footprint, as shown by Marty et al. [32] in an actualistic study of various modern lakes with mats. Thus, the variations observed in the blurred heel contours in magenta footprints could be a consequence of the posterior development of the mat.

### Trackmaker in the ecosystem

Trackmaker recognition of this abnormal and pathological trackway should be carefully considered. Small- to medium-sized avian and non-avian theropods that characterise the Las Hoyas diversity, such as the enantiornithes [69–71], and ornithomimosaur, *Pelecanimimus polyodon* [72] were discarded as putative producers due to their body size. The carcharodontosaurid, *Concavenator corcovatus* [73–74] was also discarded because it shows a pes of half the size of the magenta footprints with short and subequal toes. Interestingly, the size of the magenta footprints indicates the first record of a large theropod in the inland wetland La Huérguina ecosystem.

The presence of wrinkle structures in the #16 layer provides evidence of the palaeoenvironmental conditions of Las Hoyas. These structures have been discussed as morphological biosignatures due to the presence of mats [75], formed at the sediment-water interface, and described as the result of growth responses to physical mat destruction due to episodes of sediment reworking under flow conditions [52–76]. Accordingly, the wrinkles of Las Hoyas, arranged in a preferential orientation, would suggest a shallow pool of water with a subtle waving flow at the moment of mat fragmentation that enhanced the presence of swimming grazer fish. In this context, the dinosaur would have crossed this pool moving towards the main water source.

## Conclusions

Our challenge is to understand how and why an abnormally wide bipedal dinosaur trackway with asymmetric footprints could have been produced in the Las Hoyas ecosystem. We build a protocol with multiple techniques and scenarios led to consistent results and helped to clarify the factors necessary to explain and understand (a) the conditions of the substrate, (b) the marks resulting from animal–sediment interaction, and (c) the descriptive and comparative ichnology of dinosaurs. Thus, it is important to conclude that LH-Mg-10-16 is one of the best trackways of a theropod dinosaur, which combines a foot pathology and a fairly regular gait, although with a forced wide-trackway. Surprisingly, analogous deformities occur in modern birds despite the astonishing differences in size. The large theropod with a hip height of about 2 m would have walked on a moist mat that favoured the production of deep tracks outlining the toes. Rapid lithification is essential for the reproduction of the motion of a 5-ton dinosaur, where marks and deformations observed in the right footprint denote a greater load on the right foot, and in the left foot the toe II is backwardly directed. Biolamination of the substrate is accompanied by other mat surface structures, mostly small, thin wrinkles, which are interesting yet scarcely studied biosignatures that provide information on the palaeoenvironmental conditions. Thus, a shallow water flow was the scenario, where fish agglomerated and the theropod crossed, during the season when the microbial mat began tearing.

## Supporting information

S1 Fig Photograph. Magenta Trackway (Las Hoyas, upper Barremian, Spain) with the associated real colour of the vertex cloud.
(PNG)

S2 Fig Heat map. Mp4 Video of the of Magenta trackway (Las Hoyas, upper Barremian, Spain).
(MP4)

S1 File Alternative hypotheses. Plausible hypotheses to the deformed foot.
(DOCX)
