# Supplementary figures and images for: A theropod trackway providing evidence of a pathological foot from the exceptional locality of Las Hoyas (upper Barremian, Serranía de Cuenca, Spain)
Source: PLoS One. 2022 Apr 6;17(4):e0264406. doi: 10.1371/journal.pone.0264406 (PMC8985934; doi:10.1371/journal.pone.0264406)

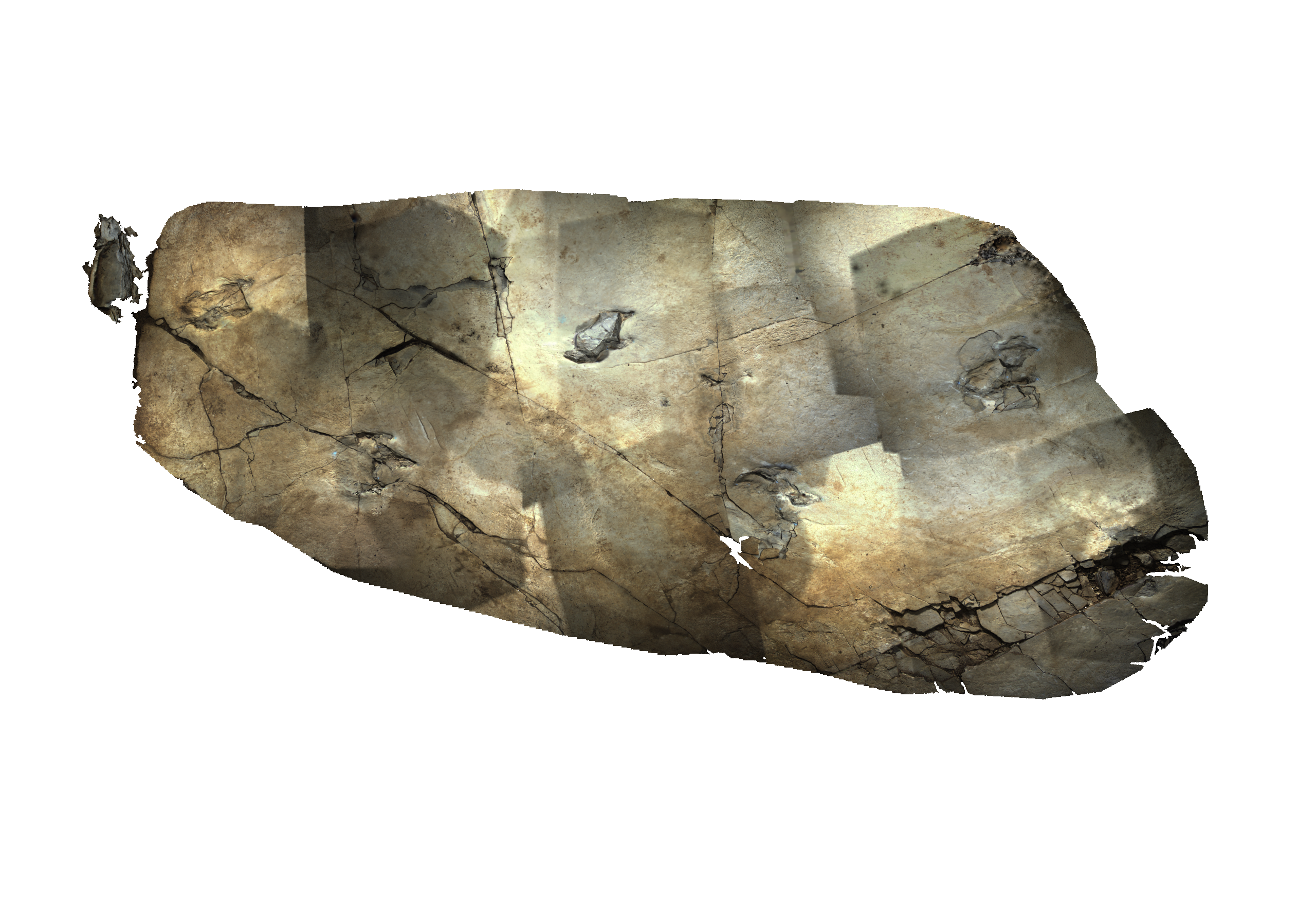

Supplement: S1 Fig — Magenta Trackway (Las Hoyas, upper Barremian, Spain) with the associated real colour of the vertex cloud. (PNG) [file pone.0264406.s001.png]
