# Supplementary material for: A theropod trackway providing evidence of a pathological foot from the exceptional locality of Las Hoyas (upper Barremian, Serranía de Cuenca, Spain)
Source: PLoS One. 2022 Apr 6;17(4):e0264406. doi: 10.1371/journal.pone.0264406 (PMC8985934; doi:10.1371/journal.pone.0264406)
Supplement: S1 File — Plausible hypotheses to the deformed foot. (DOCX) [file pone.0264406.s003.docx]

**SUPPLEMENTARY INFORMATION**

A theropod trackway providing evidence of a pathological foot from the exceptional locality of Las Hoyas (upper Barremian, Serranía de Cuenca, Spain)

Carlos M. Herrera, J. Joaquín Moratalla, Zain Belaústegui, Jesús Marugán-Lobón, Hugo Martín-Abad, Sergio M. Nebreda, Ana I. López-Archilla, Ángela D. Buscalioni

**Other plausible hypotheses to the deformed foot**

There are three plausible explanations to understand the shape of the deformed left foot (Fig .S1):

- H1: The injury of digit II. It supposes that: (a) the digit III would be the thicker but also reduced or affected in length; (b) wide interdigital areas for digits II-III and IV-III; (c) slender digit II with some phalanges lost or twisted toe, and just a splint would be preserved and impressed; (d) the pes would not be rotated and the three digits are forwardly directed; (e) stout digit IV, and (f) the preserved lateral indentation would be an artefact.
- H2: The loss of digit IV. It supposes that: (a) the digit III would be largest and longest; (b) wide interdigital areas between digits; (c) rather short digit II; (d) the observed lateral indentation posterior to digit III would follow the contour of the base of digit IV in all the left footprints; (f) the presence of a digit I impression as a rod, placed medial and posterior and connect with the “heel” foot area.
- H3: A third less plausible alternative would be an amputated digit II but a medial preserved digit I. Although in theropods the hallux tends to be backward or laterally directed, in semiplantigrade footprints, with a flat metatarsus, the digit I might be medial or forwardly oriented [1-2]. Impressions of metatarsals with traces with the hallux medial and anteriorly directed have been figured in Early Cretaceous Gondwanan trackways of large theropods [3]. In the Magenta trackway the semiplantigrade posture is discarded.

When measurements of the Magenta trackway are estimated according the hypotheses 1 and 2, the greatest differences occur in WAP and α (Table S1). Hypothesis 2 is less plausible because the left footprints would be quite rotated, and they would be stretched showing a broaden plantar surface medially, due to the incorporation of the digit I. The WAP would be even larger in this hypothesis with extreme outlier values for a theropod.

**Figure S1: Graphical representation of measurements shown in Table S1**


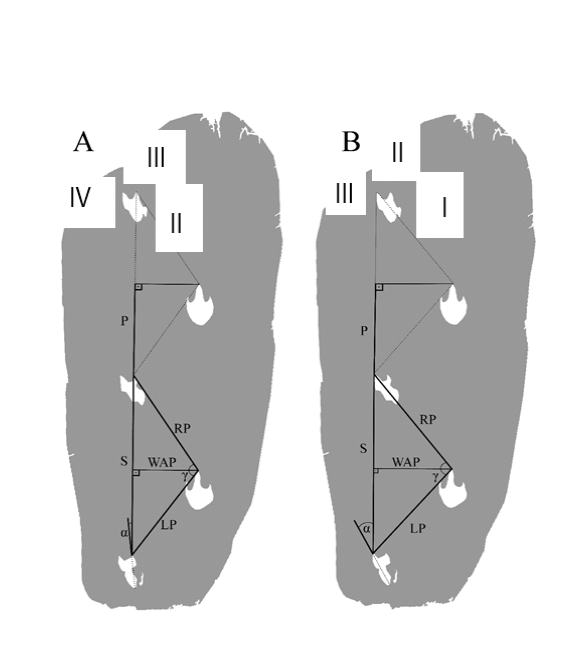


(A) Hypothesis 1( B) Hypothesis 2.

**Table S1: Measurements of the trackway according each hypothesis, based on the scanned image**.

| **Hypothesis** | **LP** | **RP** | **S** | **P** | **WAP** | **α** | **ϒ** |
| --- | --- | --- | --- | --- | --- | --- | --- |
| **H1** | 119 | 125 | 195 | 98,1 | 69,9 | 7º | 72º |
| **H2** | 126 | 130 | 192 | 98,6 | 83,6 | 33º | 77º |

H1: Hypothesis 1, digit II-deformed; H2: Hypothesis 2, loss of digit IV. Abbreviations: From digit III to digit III: LP left pace; RP: right pace; S: stride; P: progression; WAP: width of the angulation pattern (measured perpendicular to the stride length); α: rotation of the track with respect of the next stride line; ϒ: pace angulation. All length measurements in centimeters and angles in degrees.

**References**

[1] Nouri J, Díaz-Martínez, I, Pérez-Lorente F,. Tetradactyl footprints of an unknown affinity theropod dinosaur from the Upper Jurassic of Morocco. 2011, PloSOne 6, e26882. <https://doi.org/10.1371/journal.pone.0026882>.

[2] Heredia AM, Pazos PJ, Díaz-Martinez I,. Morphological variations in dinosaur trydactyl tracks from Candeleros Formation (Upper Cretaceous) of northwestern Patagonia, Argentina. 2021, Journal of South American Earth Sciences 108: 1-16.

<https://doi.org/10.1016/j.jsames.2021.103212>.

[3] Moreno K, De Valais S, Blanco N, Tomlinson AJ., Jacay J, Calvo JO.,. Large theropod dinosaur footprint associations in western Gondwana: behavioural and palaeogeopgraphic implications. 2012, Acta Palaeontologica Polonica 57(1): 73-83.
